# A New Genus and Species of Lophocateridae from Mid-Cretaceous Amber of Myanmar (Coleoptera) †

**DOI:** 10.3390/insects12121052

**Published:** 2021-11-24

**Authors:** Ya-Li Yu, Yan-Da Li, Jiří Kolibáč, Adam Ślipiński, Dong Ren, Hong Pang, Zhi-Qiang Li

**Affiliations:** 1 Guangdong Key Laboratory of Animal Conservation and Resource Utilization, Guangdong Public Laboratory of Wild Animal Conservation and Utilization, Institute of Zoology, Guangdong Academy of Sciences, Guangzhou 510260, China; yuyl@giabr.gd.cn; 2 State Key Laboratory of Biocontrol, School of Ecology, Sun Yat-sen University, Guangzhou 510275, China; lsshpang@mail.sysu.edu.cn; 3 State Key Laboratory of Palaeobiology and Stratigraphy, Nanjing Institute of Geology and Palaeontology, Chinese Academy of Sciences, Nanjing 210008, China; ydli@nigpas.ac.cn; 4 Department of Entomology, Moravian Museum, Hviezdoslavova 29a, 62700 Brno, Czech Republic; jkolibac@mzm.cz; 5 Australian National Insect Collection, CSIRO, GPO Box 1700, Canberra, ACT 2601, Australia; Adam.Slipinski@csiro.au; 6 College of Life Sciences, Capital Normal University, Beijing 100048, China; rendong@mail.cnu.edu.cn

**Keywords:** Lophocateridae, Cretaceous, Burmese amber, phylogeny

## Abstract

**Simple Summary:**

Lophocateridae are a small family of Cleroidea and their taxonomic rank has changed several times. The fossil record of Lophocateridae from Mesozoic deposits is sparse. Here, we figure and describe a new lophocaterid fossil, Gracilenticrus burmiticus, from the mid-Cretaceous amber of Myanmar. The new taxa broaden the generic and species diversity of lophocaterids and provide additional information for understanding the early evolution of Cleroidea.

**Abstract:**

A new genus and species of the cleroid family Lophocateridae are described and illustrated from the mid-Cretaceous amber of northern Myanmar. Gracilenticrus burmiticus Yu, Kolibáč & Ślipiński gen. et sp. nov. is unique among Lophocateridae in the tiny body size, frontoclypeal suture and antennal grooves absent, symmetrical antennal clubs, protrochantin reduced, tarsal claws small and widened at base. A key to the species of Mesozoic Lophocateridae is also provided. Morphological characters of the newly discovered *Gracilenticrus* were analyzed together with representatives of 43 extant genera of Cleroidea (broadly defined Trogossitidae) in a matrix of 91 characters. *Gracilenticrus burmiticus* was resolved as a member of Lophocateridae. The discovery of a diverse fauna of Lophocateridae in the mid-Cretaceous sheds a new light on the early evolution of superfamily Cleroidea.

## 1. Introduction

The family Lophocateridae are a small cosmopolitan family of cleroid beetles, comprising approximately 120 extant species in about 17 genera [1,2,3]. This group was assigned as a subfamily or tribe of Trogossitidae in previous studies [4,5,6,7,8,9]. However, the traditional broadly defined Trogossitidae are widely presumed to be paraphyly or polyphyletic by many molecular analyses [10,11,12,13,14,15], and the placement of this group is ambiguous. Recently Lophocateridae were re-elevated to the family level and revised to include *Parapeltis* and *Colydiopeltis* formerly in Peltinae: Colydiopeltini, and exclude Decamerini based on the most comprehensive molecular analysis by Gimmel et al. [1]. The tribe-level classification of the former Lophocaterinae were also shown to be somewhat problematic [1]. The revised Lophocateridae have been strongly supported as monophyletic. The genus *Grynocharis* Thomson (formerly in Lophocaterini) was placed as sister to the remainder of the family. *Parapeltis* Ślipiński, *Eronyxa* Reitter and *Trichocateres* Kolibáč occupied relatively basal positions, although their relationships were not fully resovled. The remaining sampled genera (Ancyronini and part of Lophocaterini) constitute a large clade within the family by Gimmel’s study [1].

The fossil record of Lophocateridae from Mesozoic deposits is sparse. The only three previously described members of Lophocateridae from mid-Cretaceous Burmese amber are *Burmacateres longicoxa* Kolibáč & Peris and *Parayixianteres parvus* Yu, Leschen & Ślipiński and *Zaiwa pankowskiorum* Lyubarsky et al. [16,17,18]. Here, we report another newly discovered genus based on a well preserved inclusion in the Cretaceous Burmese amber, which implies that the family was already diverse by the Cretaceous.

## 2. Material and Methods

The Burmese amber specimens studied herein originated from amber mines near Noije Bum, Hukawng Valley, Kachin State, northern Myanmar. Burmese amber is one of the most important of the fossiliferous resins from the Mesozoic, with its paleobiotic diversity reviewed by Ross et al. [19,20,21], and is renowned for yielding rich and exquisitely preserved fauna including well-preserved insects [22,23,24,25]. The age of the amber deposits is considered to be mid-Cretaceous based on U-Pb zircon dating [26,27]. The holotype of *Gracilenticrus burmiticus* sp. nov. is deposited at the Key Laboratory of Insect Evolution and Environmental Changes, Capital Normal University (CNU), Beijing, China. The amber piece was prepared using a razor table, ground with emery papers with different grit sizes and finally polished with polishing powder.

The specimen was examined under a Leica M205C stereomicroscope (Leica, Wetzlar, Germany). Photographs under incident light were mainly taken with a Canon EOS 5D Mark II camera and a MP-E 65mm f/2.8 1-5X Macro lens (Canon, Tokyo, Japan). Widefield fluorescence images were captured with a Zeiss Axio Imager 2 light microscope combined with a fluorescence imaging system (Zeiss, Jena, Germany). Confocal images were obtained with a Zeiss LSM710 confocal laser scanning microscope, using the 488 nm Argon laser excitation line (Zeiss, Jena, Germany). Images under incident light and widefield fluorescence were stacked in Helicon Focus 7.0.2 or Zerene Stacker 1.04. Confocal images were stacked with color coding for depth in ZEN 2.3 (Blue Edition). Images were further processed in Adobe Photoshop CS6 to enhance contrast.

The morphological terminology follows Lawrence et al. [28] and the classification of families and subfamilies of Cleroidea follows Lawrence et al. [29] with amendments by Gimmel et al. [1].

**Figure 1:**
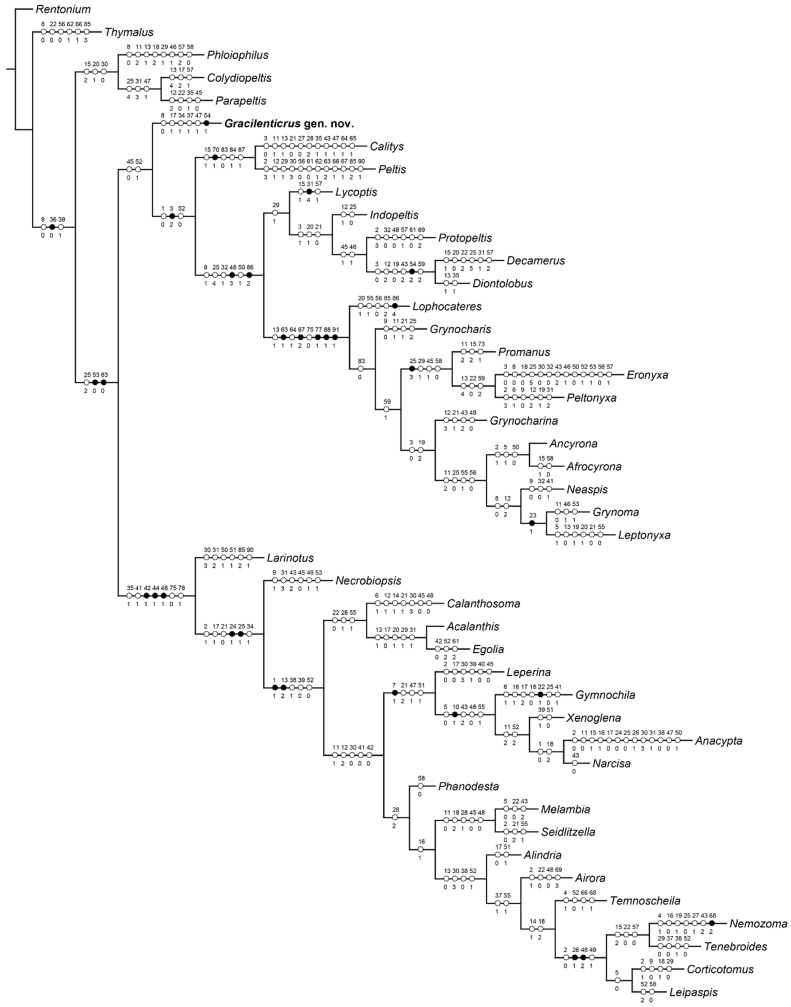
The most parsimonious tree showing the placement of *Gracilenticrus burmiticus* gen. et sp. nov. within the traditional Trogossitidae (length = 501; CI = 29; RI = 58). Only unambiguous character changes are mapped (unique and homoplastic features are shown as black or white circles, respectively).

To evaluate the systematic placement of the new genus, a morphological phylogenetic analysis was performed. The data matrix (Supplemental Material Datas S1–S3) was derived from a previously published dataset [30,31], and includes 91 morphological characters of both adults and larvae. *Gracilenticrus* gen. nov. was analyzed together with representatives of 43 extant genera of the traditional broadly defined Trogossitidae. *Rentonium* Crowson (now classified in family Rentoniidae) was selected as the outgroup taxon. The assembled data matrix was analyzed by TNT v. 1.5 software [32] under parsimony, using the implicit enumeration algorithm criterion with all characters unordered. Parsimony analysis using implied weighting (*K* = 12, as recommended by Goloboff et al. [33] and Smith [34]) yielded a single most parsimonious tree with a step length of 501, a consistency index (CI) of 0.29, and a retention index (RI) of 0.58 (Figure 1).

## 3. Results

### 3.1. Systematic Palaeontology

Order Coleoptera Linnaeus, 1758

Suborder Polyphaga Emery, 1886

Superfamily Cleroidea Latreille, 1802

Family Lophocateridae Crowson, 1964

**Genus***Gracilenticrus***Yu, Kolibáč & Ślipiński gen. nov**.

Type species *Gracilenticrus burmiticus* Yu, Kolibáč & Ślipiński sp. nov.

**Etymology.** The generic name is a combination of the latinized adjective “*gracilentus*” (meaning slender) and “*crus*” (meaning leg), referring to its relatively slender legs. The gender of the name is masculine. 

**Diagnosis.** Body minute, weakly convex, elongate-oval. Frontoclypeal suture and antennal grooves absent. Eyes moderately large. Antennae with 11 antennomeres, club large and symmetrical. Pronotal disc distinctly wider than long, weakly convex in center, conspicuously but sparsely granulate. Prosternal process narrow and acute apically. Procoxal cavities broadly open externally. Protrochantin reduced. Mesocoxal cavities open. Mesocoxae narrowly separated. Elytral epipleura wide along humerus, continuously contracting towards apex. Each elytron with 5 narrow and dark costae. Protibia with moderate and slender spines along edge. Claws small, widened at base.

**Figure 2:**
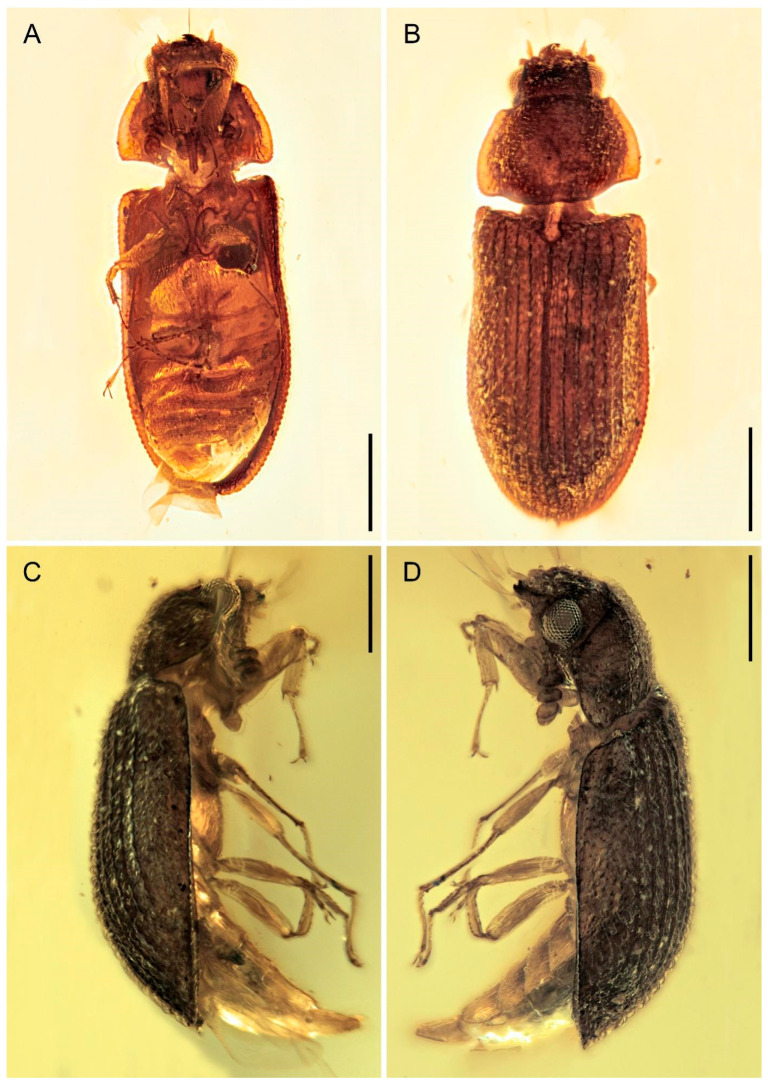
Photograph of *Gracilenticrus burmiticus* gen. et sp. nov. in Upper Cretaceous amber from Myanmar, holotype (CNU-COL-MA2021001), under incident light. (**A**) Ventral view. (**B**) Dorsal view. (**C**,**D**) Lateral view. Scale bars = 0.5 mm.

**Figure 3:**
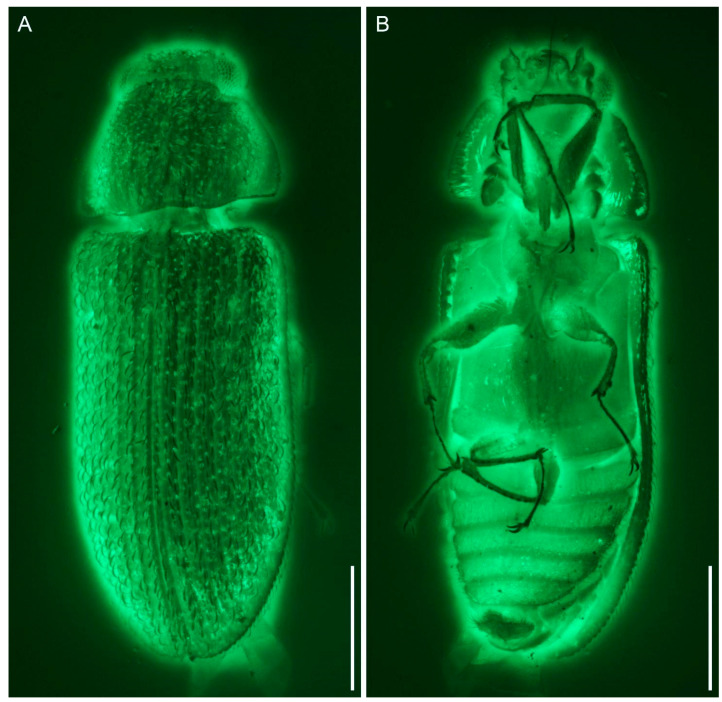
Photograph of *Gracilenticrus burmiticus* gen. et sp. nov. in Upper Cretaceous amber from Myanmar, holotype (CNU-COL-MA2021001), under widefield fluorescence. (**A**) Dorsal view. (**B**) Ventral view. Scale bars = 0.5 mm.

**Figure 4:**
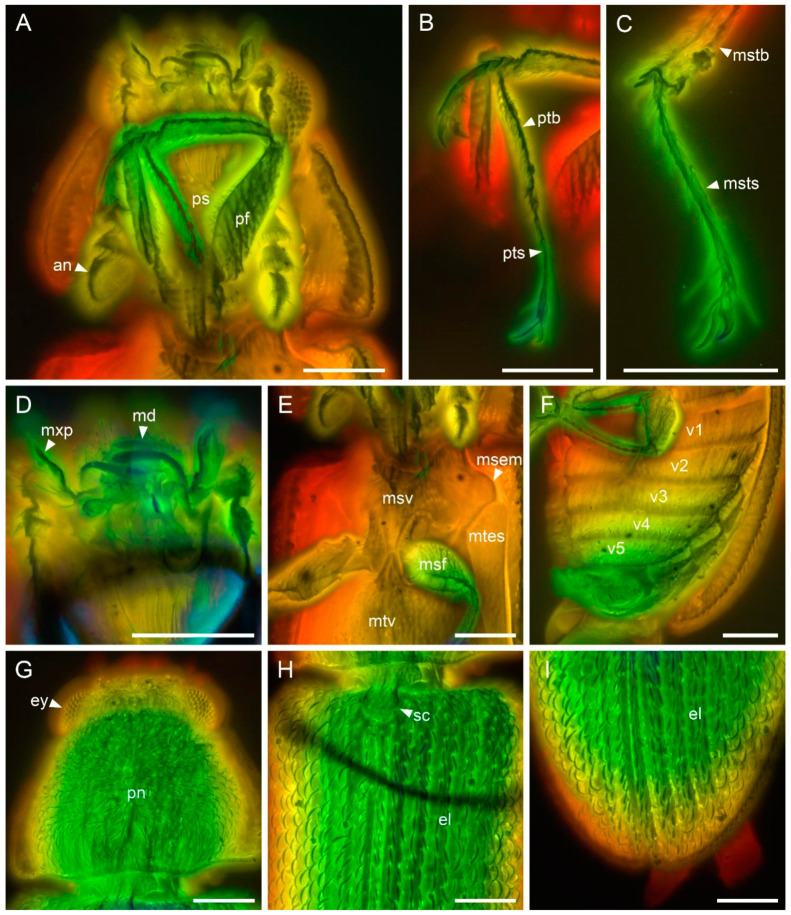
Details of *Gracilenticrus burmiticus* gen. et sp. nov., under confocal microscopy, with depth color coding. Dorsal views in (**G**–**I**), ventral views in (**A**–**F**). (**A**) Head and prothorax. (**B**) Protibiae and -tarsi. (**C**) Left mesotarsus. (**D**) Mouthparts. (**E**) Mesothorax. (**F**) Abdomen. (**G**) Head and prothorax. (**H**) Anterior portion of elytra. (**I**) Posterior portion of elytra. Abbreviations: an, antenna; el, elytron; ey, compound eye; md, mandible; msem, mesepimeron; mstb, mesotibia; msf, mesofemur; msts, mesotarsus; msv, mesoventrite; mtes, metanepisternum; mxp, maxillary palp; pf, profemur; pn, pronotum; ps, prosternum; pts, protarsus; ptb, protibia; sc, scutellum; v1–5, ventrites 1–5. Scale bars: 200 μm.

*Gracilenticrus burmiticus* Yu, Kolibáč & Ślipiński, sp. nov. (Figure 2, Figure 3 and Figure 4).

**Etymology.** The specific name *burmiticus* refers to the occurrence of the fossil in burmite (Burmese amber). 

**Material.** Holotype, CNU-COL-MA2021001; lowermost Cenomanian, Hukawng Valley, northern Myanmar; deposited in the Key Laboratory of Insect Evolution & Environmental Changes, Capital Normal University in Beijing, China.

**Measurements.** Holotype: body length 2.5 mm, pronotum length 0.6 mm, elytra length 1.6 mm, pronotum width 0.9 mm, elytra width (together) 1.0 mm, antenna length 0.6 mm.

**Diagnosis.** As for the genus.

**Description (based on holotype).** Body weakly convex, elongate-oval, with dense pubescence composed of inclined short setae, lateral margins of pronotum broadly explanate, elytra narrowly explanate.

Head prognathous, transverse; sculpture of dorsal surface formed by inclined setae. Frons simple, without longitudinal groove or horn-like processes. Frontoclypeal suture absent. Eyes moderately large, not emarginated, lateral and elevated, very coarsely faceted, without interfacetal setae; frontal space between eyes about twice eye diameter. Antennal insertions widely separated. Subantennal groove absent. Antennae 11-segmented, with very large 3-segmented symmetrical relatively loose club; scape and pedicel robust; terminal antennomere broadly rounded. Mandibles with two apical teeth. Maxillary palps 4-segmented; palpomere 1 small; palpomeres 2–3 smilar in size; apical palpomere elongate. Labrum not fused. Ligula rigid, weakly emarginate, not retroflex. Labial palps 3-segmented. Gular sutures not observed. Mentum trapezoidal. Submentum without tuft of setae.

Prothorax transverse, about 0.67 times as long as wide, widest along base, narrowed anteriorly; anterior angles very slightly prominent, blunt; posterior ones also blunt; lateral carinae complete, slightly crenulate; anterior edge and posterior edge slightly produced at middle, sinuate. Pronotal disc weakly convex; conspicuously but sparsely granulate. Procoxal cavities transverse, broadly open externally; prosternal process narrow, parallel, extending well behind procoxae, acute apically with a longitudinal carinae in the middle. Procoxae conspicuously transverse, weakly projecting; protrochantin reduced. Mesoventrite wide and transverse, slightly convex; mesepimeron and mesanepisternum triangular. Mesocoxal cavities slightly oblique and open to mesepimeron. Mesocoxae nearly spherical and narrowly separated, slightly projecting. Metaventrite wide, with complete discrimen, metanepisternum broad. Metacoxae large, flat and extending laterally to meet elytra.

Scutellum weakly transverse, rounded apically. Elytra 1.60 times as long as width combined, 2.67 times as long as pronotum length, oblong, approximately parallel-sided (weakly narrowed at basal third), completely covering abdomen, their humeral portion subequal with pronotal base. Epipleuron moderately broad and apparently complete to apex, widest at humeral portion, gradually narrowed apically. Each elytron with 5 narrow and black costae separated by two rows of punctures bearing posteriorly inclined, somewhat squamiform setae; each costa bears setae similar to those on interstrial punctures; lateral portion of elytra beyond last costa with dense and rather irregular setiferous punctures.

Legs relatively slender, only femur robust and clavate. Tibiae with one row of spines along outer edge; protibial apex with single hooked spur; meso- and metatibiae with paired apical spurs. Tarsi 5-5-5, apical tarsomere as long as combined length of tarsomeres 1–4; claws small, widened at base.

Abdomen with five ventrites; intercoxal process narrowly rounded apically; ventrites more or less equal in length.

### 3.2. Phylogenetic Results

Previous studies have shown that morphological information alone is not sufficient to solve the phylogeny of traditional trogossitid taxa [1,31]. However, the phylogenetic analysis may still provide some helpful information for the placement of our fossil. In our analysis, the newly discovered fossil, *Gracilenticrus burmiticus*, was nested within a clade comprising *Peltis*, *Calitys* and traditional Lophocaterinae. This clade contains all of the modern Lophocateridae defined by Gimmel et al. [1] except for *Colydiopeltis* and *Parapeltis*. Within the clade, the following relationships were recovered: (i) the genus *Gracilenticrus* gen. nov. was placed as sister to the remainder of the clade; (ii) *Calitys* (Trogossitidae: Calitynae) and *Peltis* (Peltidae) formed a grade of early branches; (iii) the remaining part of this clade was split into two groups, with the first group including genera from Lophocateridae, Protopeltidae and Thymalidae, and the second group consisting of eleven Lophocateridae genera. Unfortunately, most terminals in this clade, including *Gracilenticrus*, have large number of characters coded as uncertain because critical parts were not preserved and larval characters have not been observed. As a result, the position of *Gracilenticrus* in this cladogram is only tentative and cannot be regarded as firmly solved.

## 4. Discussion

Although the placement of *Gracilenticrus* is not firmly solved by the phylogenetic analysis, it is apparent that *Gracilenticrus* is closely related to the Lophocateridae group. Based on the detailed examination of morphological characters, the genus can be unequivocally assigned to Lophocateridae. As a member of the Lophocateridae, *Gracilenticrus* has the weakly convex body with explanate sides, the strongly transverse procoxae, open procoxal cavities and laterally expanded metacoxae reaching the elytral epipleura. On the other hand, *Gracilenticrus* differs from all other members of Lophocateridae in the absence of frontoclypeal suture, eyes moderate, antennal club symmetrical and protibia with robust spines along edge.

While most of the lophocaterid genera including *Ancyrona*, *Antillipeltis*, *Colydiopeltis*, *Grynocharina*, *Grynoma*, *Leptonyxa*, *Lycoptis*, *Neaspis*, *Parapeltis*, *Peltonyxa* have 10 or fewer antennomeres, *Gracilenticrus* has 11-segmented antennae. Based on the characters of anterior pronotal angles and elytra, we can easily distinguish *Gracilenticrus* from *Afrocyrona, Indopeltis* and *Eronyxa*, since *Afrocyrona* has a distinctly produced forward anterior pronotal angles and elytral scales present; *Indopeltis* has acute anterior pronotal angles and each elytron having six carinae that are interrupted; and *Eronyxa* has the anterior pronotal angles not produced forward and elytral carinae reduced. The new genus differs from *Trichocateres* in the lack of two sharp grooves on the prosternal process and upper surfaces without tufts of long and light-colored hairs. *Gracilenticrus* is also different from *Grynocharis* in the absence of antennal grooves, smaller body size, slightly crenulate lateral pronotal carinae (simple in *Grynocharis*) and each elytron having five carinae (six in *Grynocharis*). The new genus can be easily distinguished from *Lophocateres* due to the flat body shape, presence of antennal grooves, absence of denticle on claws, and each elytron with seven carinae of the latter. The new genus seems to be most similar to *Promanus* in appearance, but the latter has a larger body size and flat body shape, with antennal grooves present and denticles on claws absent. With respect to fossils, *Gracilenticrus* can be distinguished from *Sinosoronia* Zhang, *Cretamerus* Peris, Kolibáč & Delclòs and *Promanodes* Kolibáč, Schmied, Wappler & Kubisz by having antennae 11-segmented, elytra with distinct longitudinal carinae and regular punctures, and abdomen with five ventrites [35,36,37]. *Gracilenticrus* also differs from *Yixianteres* Yu, Ślipiński, Leschen, Ren & Pang and *Paracretocateres* Yu, Ślipiński, Leschen, Ren & Pang in having a smaller and setose body [38]. The previously reported lophocaterid genera from Burmese amber, *Parayixianteres* Yu, Leschen & Ślipiński and *Burmacateres* Kolibáč & Peris, differ distinctly from *Gracilenticrus* in having relatively flattened body, frontoclypeal suture present, slightly asymmetrical antennal club, strongly prominent forward anterior angles and exposed protrochantins. Another lophocaterid genus known from Burmese amber, *Zaiwa*, has 10-segmented antennae with apical segment more than twice as long as the preceding two, pronotum about 1.8 times as wide as long and elytral without costae, and therefore can be easily distinguished from *Gracilenticrus*. A key of the Mesozoic Lophocateridae is provided in Appendix A.

Previous studies suggest that the more primitive cleroids were mainly fungus-feeding and predatory followed by later shifts to flower-feeding [1,39]. The weakly convex body form of *Gracilenticrus* indicates it is probably not fungivorous. Since distinctly erect vestiture is mostly known in predatory surface dwellers in Cleroidea [16], we speculate the habits of this new genus also predatory.

## 5. Conclusions

The Mesozoic taxon *Gracilenticrus* Yu, Kolibáč & Ślipiński shares many important characters with the extant lophocaterids to support its placement in Lophocateridae. Our discovery of this new genus and new species from the mid-Cretaceous amber of northern Myanmar reflect an already strong diversification of the family during the Cretaceous period.

## Data Availability

The original confocal data are available in Zenodo repository (https://doi.org/10.5281/zenodo.5574817).

## Supplementary Materials

The following are available online at https://www.mdpi.com/article/10.3390/insects12121052/s1: Data S1: List of characters used in the phylogenetic analysis; Data S2: Morphological dataset used for the analysis in XLSX format; Data S3: Morphological dataset used for the analysis in TNT format.

## Appendix A. Key to the Mesozoic Lophocateridae

(*Sinosoronia longiantennata* Zhang is omitted in the key due to the poor preservation and limited description).

1. Pronotum strongly transverse (more than 2.5 times as wide as long)……………………………………………………………*Ancyrona eocenica* Schmied et al.
  Pronotum slightly or moderately transverse (less than 2.5 times as wide as long)………………………………………………………………………………………………2
2. Antennae 9-segmented………………………………………………………………………3
  Antennae 10-segmented or 11-segmented…………………………………………………4
3. Pronotum sides distinctly explanate with distinctly, coarsely crenulate edges; pronotal punctures unevenly distributed but usually separated by one or two diameters; length 3 mm.…………………………………………*Antillipeltis iviei* Lawrence, Leschen & Ślipiński
  Pronotum sides slightly explanate with weakly crenulate edges; pronotal punctures usually separated by more than two diameters; 2.7 mm …………*Antillipeltis alleni* Lawrence, Leschen & Ślipiński
4. Apical antennal segment more than twice as long as the preceding two……*Zaiwa pankowskiorum* Lyubarsky et al.
  Apical antennal segment less than twice as long as the preceding two………………5
5. Antennae 10-segmented……………………………………………………………………6
  Antennae 11-segmented……………………………………………………………………8
6. Elytra with distinct carinae…………………………………………………………………7
  Elytra without carinae…………………………………………*Cretamerus vulloi* Peris et al.
7. Head (sub)glabrous; pronotum and elytra pubescent along margins; length 5 mm …………………………………………………………*Promanodes serafini* Kolibáč et al.
  Head, pronotum and elytra with distinct pubescence; length 3 mm……*Promanodes alleni* Kolibáč
8. Body clothed with distinct hairs or setae…………………………………………………9
  Body (sub)glabrous…………………………………………………………………………10
9. Frontoclypeal suture present; antennal club slightly asymmetrical; anterior pronotal corners moderately projecting; protrochantins exposed……………*Burmacateres longicoxa* Kolibáč & Peris
  Frontoclypeal suture absent; antennal club slightly symmetrical; anterior pronotal angles very slightly prominent, blunt; protrochantin reduced……………*Gracilenticrus burmiticus* Yu, Kolibáč & Ślipiński
10. Anterior angles somewhat prominent……………………*Paracretocateres bellus* Yu et al.
  Anterior angles strongly prominent forward……………………………………………11
11. Prosternal process broadly angulate at level of procoxae, with short and rounded apex extending beyond coxae; length 3.9 mm………………*Yixianteres beipiaoensis* Yu et al.
  Prosternal process parallel-sided, relatively broad, straight or slightly expanding apically; length 28.3 mm………………………*Parayixianteres parvus* Yu, Leschen & Ślipiński
