# Supplementary material for: A New Genus and Species of Lophocateridae from Mid-Cretaceous Amber of Myanmar (Coleoptera)
Source: Insects. 2021 Nov 24;12(12):1052. doi: 10.3390/insects12121052 (PMC8709423; doi:10.3390/insects12121052)
Supplement: Supplementary file 1 [file insects-12-01052-s001.zip › Data S1.rtf]

File S1. List of characters used in the phylogenetic analyses (adapted from Kolibáč, 2008 and Li et al., 2021). All characters were treated as non-additive. None of the characters were deactivated. For a explanation of the characters, please refer to Kolibáč (2008).

Adult characters
01. Body shape: flat, oval = 0; elongate = 1; oval, weakly convex (not conglobate) = 2; oval, conglobate = 3.
02. Gular sutures: widely separated at base, convergent at apex = 0; narrowly separated at base, subparallel = 1; reduced in length = 2; widely separated at base, subparallel = 3.
03. Frontoclypeal suture: present, straight = 0; absent = 1; broadly emarginate (curved posteriad) = 2.
04. Frons – longitudinal groove or depression: absent = 0; present = 1.
05. Cranium in ventral view – lateral tufts of long setae: absent = 0; present = 1.
06. Submentum of males – tuft of setae: absent = 0; present = 1.
07. Submentum – anterior margin with row of setae: absent = 0; present = 1.
08. Antennal groove: absent = 0; present = 1.
09. Eyes – size: moderate (space between eyes about twice eye diameter) = 0; large (space between eyes narrower than eye diameter), eyes laterally situated = 1; large (space between eyes
narrower than eye diameter), eyes dorsolaterally situated = 2; small and flat (space between eyes more than twice eye diameter) = 3.
10. Eyes – number: 2 = 0; 4 = 1.
11. Epicranial acumination: moderate = 0; deep = 1; absent = 2.
12. Lacinia – number of hook-like spines at apex: 2 = 0; 1 = 1; 0 = 2; 3 = 3.
13. Galea – shape: elongate = 0; subclavate = 1; clavate = 2; partially fused with lacinia = 3; very small = 4.
14. Galea – ciliate setae: absent = 0; present = 1.
15. Mediostipes – Lacinia: not fused = 0; partially fused = 1; completely fused = 2.
16. Palpifer – outer edge: smooth = 0; denticulate = 1.
17. Mandible – number of apical teeth: 2 teeth situated in horizontal axis = 0; 2 teeth situated in vertical axis = 1; 1 tooth present = 2.
18. Mandibular mola: present = 0; reduced but remnant of mola present = 1; absent = 2.
19. Mandible – penicillus (at mandibular base): membrane with fine pubescence = 0; membrane and pubescence absent = 1; only long setae present, membrane absent = 2.
20. Mandible – pubescence above mola or cutting edge (prostheca): absent = 0; present = 1. 
21. Mandible – ventral ciliate furrow: furrow ciliate = 0; furrow not ciliate = 1; absent = 2.
22. Mandible – basal notch: moderate = 0; deep (reaching about 1/3 of mandible) = 1; shallow or absent = 2.
23. Labrum – Cranium: not fused = 0; fused = 1.
24. Labrum – sclerite in epipharynx: absent = 0; present = 1.
25. Labrum – projection of lateral tormal process: projection not developed (all remaining genera) = 0; curved downwards, processes not connected (Airora) = 1; curved downwards, processes with bridge (Peltis) = 2; projection reduced or absent (Promanus) = 3; projection curved upwards (Colydiopeltis) = 4; projections extending laterally and downwards (Eronyxa) = 5.
26. Ligula – ciliate setae: absent = 0; present = 1.
27. Ligula: rigid = 0; membranous = 1.
28. Ligula: not retroflex = 0; weakly retroflex = 1; strongly retroflex = 2.
29. Ligula – shape: weakly emarginate = 0; deeply emarginate = 1.
30. Labium – hypopharyngeal sclerite: absent = 0; sickle-shaped = 1; H-shaped = 2; 2 separate sclerites = 3.
31. Antenna: 11-segmented = 0; 10-segmented = 1; 9-segmented = 2; 8-egmented = 3; 7-segmented = 4.
32. Antennal club: antennomeres symmetrical = 0; antennomeres asymmetrical = 1;
33. Antennal club: weakly asymmetrical (one to three terminal antennomeres oval, outer side of antennomere narrowed) = 0; distinctly asymmetrical (one to three terminal antennomeres almost triangular, outer side of antennomere not narrowed) = 1.
34. Antenna – sensorial fields: absent = 0; present = 1.
35. Procoxal cavities externally: open = 0; closed = 1.
36. Procoxal cavities internally: open = 0; closed = 1.
37. Pronotum – shape: distinctly wider than long = 0; as wide as long or longer than wide = 1.
38. Pronotum – shape: not cordate = 0; cordate (narrowed towards base) = 1.
39. Mesothorax – prepectus: present = 0; absent = 1.
40. Mesocoxal cavities: open = 0; closed = 1.
41. Mesocoxal cavities: narrowly separated (space between coxae narrower than half of transverse diameter of coxa) = 0; widely separated (space between coxae approx. as wide as half of transverse coxal diameter) = 1.
State 1 occurs only in the fossil Microtrogossita.
42. Elytra – long hairs: absent = 0; present = 1.
43. Elytral epipleure: moderate (wide along humerus, continuously contracting towards apex) = 0; wide (same width along whole length) = 1; thin along whole length beyond humerus = 2.
44. Elytral lock: absent = 0; present = 1.
45. Elytra – carinae: conspicuous = 0; inconspicuous (absent) = 1. 
46. Elytra – punctation: regular, in rows = 0; irregular = 1.
47. Elytra – scales: absent = 0; present = 1. 
48. Wing – radial cell: oblong (or reduced) = 0; triangular = 1; open (outer vein present) = 2; cell moved down, often small = 3.
49. Wing – wedge cell: present = 0; absent = 1; small (Peltis) = 2. 
50. Wing – cross vein MP3-4: present = 0; absent = 1. 
51. Wing – cross vein AA1+2-3+4: absent = 0; present = 1.
52. Protibiae – spines along side: moderate, slender (spines fine, much longer than wide at base) = 0; large (spine robust, about as long as width at base) = 1; reduced (absent) = 2.
53. Protibiae – hooked spur: present (well-developed) = 0; absent (apical spurs fine, not hooked) = 1.
54. Tarsal claws – denticle: absent = 0; small (only base of claw roundly thickened) = 1; well-developed (claw with distinct denticle) = 2.
55. Parasternites in abdominal sternites III-VII: absent = 0; one = 1; two = 2.
56. Male abdominal sternite VIII - spiculum gastrale: absent = 0; present = 1. 
57. Aedeagus – number of parts of tegmen: 3 parts = 0; 2 parts = 1; 1 part = 2.
58. Ovipositor – coxitae: divided = 0; undivided = 1. 
59. Biology: fungivorous = 0; predatory = 1; floricolous = 2.

Larval characters
60. Cranium – frontal arms: strongly curved (cucujoid) = 0; weakly curved (e.g. Larinotus) = 1; V-shaped = 2; Y-shaped = 3.
61. Cranium – epicranial stem: present = 0; reduced (shorter than 1/5 of cranium) = 1; absent = 2. 
62. Cranium – endocarina: present = 0; absent = 1.
63. Cranium – gular sutures: conspicuous, parallel = 0; conspicuous, convergent = 1; reduced, inconspicuous = 2.
64. Gula – anterior apodemes: present = 0; absent = 1.
65. Cranium – paragular sclerites: absent = 0; present = 1.
66. Cranium – hypostomal rods: absent = 0; present = 1.
67. Cranium – stemmata: 5 = 0; 3 = 1; 2 = 2.
68. Mandible – apical teeth: two, horizontally situated = 0; two, horizontally and vertically situated = 1; one tooth = 2.
69. Mandible – prostheca (lacinia mandibulae): several small spines = 0; plumose = 1; tridentate = 2; absent = 3.
70. Mandibular mola: present = 0; reduced = 1; absent = 2.
71. Maxillary palpi: 3-segmented = 0; 2-segmented = 1.
72. Palpifer: present = 0; absent = 1.
73. Lacinia or mala – pedunculate seta: absent = 0; present = 1.
74. Lacinia or mala: simple = 0; bilobed = 1.
75. Lacinia or mala – apical spines or bidentate protrusion: absent = 0; present = 1.
76. Cardo – stipes: not fused = 0; partially fused = 1.
77. Cardo: much smaller than stipes = 0; nearly as large as stipes = 1.
78. Ligula: present = 0; absent = 1.
79. Labial palpi: 2-segmented = 0; 1-segmented = 1.
80. Prementum: in single part = 0; in two parts = 1.
81. Prementum – anterior margin: even = 0; with notch = 1; projecting = 2.
82. Labrum – torma: single compact plate = 0; two separate lateral sclerites = 1; H-shaped = 2.
83. Antenna: joints 1, 2 elongate = 0; joints 1, 2 transverse = 1; 1st transverse, 2nd elongate = 2.
84. Antenna – sensory appendix: medium-sized (to half of joint 3) = 0; very small = 1; longer than half of joint 3 = 2.
85. Thoracic sclerites, pattern (dorsally): 1-2-2 = 0; 1-0-0 = 1; 0+0+0 = 2; 2-0-0 = 3.
86. Thoracic sclerites, pattern (ventrally): 3+1+1 = 0; 1+1+1 = 1; 1+0+0 = 2; 0+0+0 = 3; 2+0+0 = 4.
87. Trochanter: triangular = 0; oblong = 1.
88. Abdominal segment IX: not divided = 0; transversely divided = 1.
89. Abdominal tergite IX: flat = 0; depressed (concave) = 1.
90. Urogomphi: present, hooked = 0; minute = 1.
91. Urogomphi – median process: absent = 0; present = 1.
